# The Origin of a Trans-Generational Organization in the Phenomenon of Biogenesis

**DOI:** 10.3389/fphys.2019.01222

**Published:** 2019-09-24

**Authors:** Alvaro Moreno

**Affiliations:** Department of Logic and Philosophy of Science, IAS-Research Centre for Life, Mind and Society, University of the Basque Country (UPV/EHU), San Sebastian, Spain

**Keywords:** biogenesis, organization, (self)reproduction, individual-collective duality, trans-generational causal entailments

## Abstract

One of the central issues of the whole process of biogenesis is how to understand the progressive constitution of a large (in spatial and temporal terms) system that transcends the individual sphere of proto-metabolic organizations and includes collective networks, both synchronous (i.e., proto-ecosystem webs) and asynchronous (i.e., trans-generational protocell populations). This paper analyzes the appearance of a minimal form of reproduction in the process of biogenesis from an organizational perspective. This perspective highlights the problem of how a process transcending the actual organization of the reproducing entities (i.e., protocells) could have a causal power. It is proposed that this problem may be explained if we consider that reproduction generates a kind of feedback between the actual concatenation of the processes of each reproducing cycle and the type continuity that a reliable iteration of these cycles creates. Thus, reproduction generates a new form of self-maintaining system linking “organismal” and “evolutionary” domains, since the consequence of the iteration of self-reproducing cycles is the long-term continuity of a specific type of SM compartmentalized organization, and the functional role of a particular self-reproducing organization (token) lies in its capacity to trigger a diachronic succession of similar self-reproducing organizations, i.e., a lineage.

## Introduction

Physiology is usually understood as the study of the (current) organization of a living system, or in other words, the study of its mechanisms and functions. It is normally implicitly understood that the term “living system” refers to a cohesive and individuated entity, namely an *organism*. As stated by Widmaier et al. (2016), physiology deals with fundamental biophysical and biochemical phenomena, or in simpler terms, the control mechanisms that constitute organisms’ actual organization. The evolutionary perspective, on the other hand, deals with changes in the hereditary features of certain collections of organisms (termed *populations*) over successive generations.

However, as we shall argue, looking at this issue from the perspective of its origins makes it easier to understand the connection between the “physiological” and the “evolutionary” dimensions of the phenomenon of life. This is because one of the first things to consider when we address the question of the origin of biological organization is the fact that the appearance of full-fledged living systems was necessarily the result of a historical process. Only through a historical process could those rare changes and variations (particularly functional innovations) which occurred in the actual (and ephemeral) organization of earlier individuated systems have been preserved and accumulated, thus enabling a gradual and ongoing increase in complexity.

Life itself is, of course, an example of an actual organization, but because this organization is so complex, it cannot have appeared spontaneously: high complexity cannot emerge “from scratch”. Consequently, the process by which physico-chemical systems have managed to generate increasingly complex systems, capable somehow of retaining their acquired complexity, should be viewed as an entailed process of accumulative inventions. Hence, the appearance of life is, also, a historical process: each prebiotic entity, characterized by an actual organization, is only possible due to a set of inherited factors, which include everything ranging from internally inherited components (“genes”) to collectively built environmental conditions (“ecological niches”). The study of the origins of life is therefore the study not only of how sets of chemicals have created localized and cohesive forms of self-maintaining organizations (“proto-organisms”), but also the study of how they have reproduced to enable the emergence of lineages and historical changes. It is the study of how sets of these proto-organisms have progressively changed their local environments, and how in turn these changes have facilitated the appearance of more complex proto-organisms. In sum, the question of how the connection between current organization and historicity has appeared and developed seems of paramount importance. In other words, to explain the origin of life, we must explain how a form of organization that was both sufficiently simple so as not to require any form of historical inheritance, but complex enough to generate an entailed process of accumulative inventions, namely, a historical process, could have appeared. Hence, this process (called “prebiotic evolution”) requires as its starting point the appearance of a relatively complex organizational threshold, given that several non-trivial conditions would have to have been met.

There are two aspects to prebiotic evolution. One is the emergence of spatially bounded forms of organization, capable (at least in a minimal sense) of self-maintenance. The other is the development of a mechanism ensuring the accumulative retention of the organizational and structural innovations generated within these localized systems, beyond their presumably ephemeral lifespan. This mechanism was reproduction. This is why reproduction is probably one of the most salient milestones in biogenesis. Yet, the two aspects are really two sides of the same coin, since reproduction is a form of organization that copies itself and, in consequence, is merely a very specific way of self-maintaining an organization, through propagation. Seen from the most generic perspective, reproduction is a consequence of the way in which a system (or two systems, if reproduction is sexual) is organized; and since (reliable) reproduction leads to lineages and historical changes, the question of how the connection between the current organization and reproduction emerged and developed in early proto-organisms seems of paramount importance.

For these reasons, in this paper, we will analyze how the appearance and evolution of reproduction provides insight into the progressive constitution of a large (in spatial and temporal terms) system that transcends the individual sphere of proto-metabolic organizations and includes asynchronous (i.e., trans-generational protocell populations) and collective networks which are both synchronous (i.e., proto-ecosystem webs) in nature. As we shall argue, this approach will enable us to develop an organizational account that may help bridge the gap between the physiological and evolutionary research traditions.

The paper is organized as follows. First, we will analyze which kind of system could be a candidate for initiating a process of chemical complexification leading to life, in the form of self-maintaining compartmentalized organizations. Next, we will discuss how this initial organizational complexity could have been retained, along with the role played by an early form of reproduction in the creation of an entailed process of complexification beyond the “lifespan” of the aforementioned compartmentalized forms of organization. The emergence of reproduction raises many conceptual paradoxes, which will be discussed in the Section “The Paradoxical Nature of Reproduction.” Finally, in the Section “The Consequences of Reproduction: The Appearance of a Multidimensional Organization,” we will show how, as a consequence of earlier forms of reproduction, prebiotic evolution unfolded the progressive constitution of a multi-scale system, consisting of one domain with individuated proto-metabolic organizations, and another with a large (in spatial and temporal terms) system of ecological networks.

## The Origin Problem: What is the Starting Point of Prebiotic Evolution?

The origins of prebiotic evolution are probably plural. One of the initial paths for the origin of life is, of course, the complexification of chemical processes, which occurred in concrete places under very specific boundary conditions. At first, relatively simple systems could have appeared spontaneously, and their persistence was ensured because, given the favorable environmental conditions, they could easily have arisen spontaneously time and time again. Hence, one obvious requirement for prebiotic evolution is that we must start with systems which, in principle, were sufficiently simple so as to have emerged in a spontaneous manner from a series of material aggregates under the conditions which existed at the time of the primitive Earth. Consequently, a series of highly specific boundary conditions must be envisaged (see, for example, Nisbert and Sleep, 2001) that could lead, on the one hand, to the production of new and more complex compounds, endowed with catalytic or template properties; and on the other, to the generation of far-from equilibrium self-maintaining (SM) reaction networks. However, such systems most likely came after many other systems, the maintenance of which basically depended on boundary conditions that, in terms of complexity, were much more advanced than they themselves^1^.

In certain places and under very specific boundary conditions, different sets of reactions may give rise to increasingly complex cyclic processes, leading to the appearance of increasingly complex *self-maintaining* networks (Martin and Russell, 2003). The core of a SM network is an autocatalytic reaction loop. An autocatalytic reaction loop arises as a result of the exploration of the structure of a chemical environment and its starting components’ reactivity space, connecting intermediate products (substrates rather than catalysts) in a cyclic pathway. Yet, in an autocatalytic cycle, the reactions are controlled kinetically, i.e., are prompted by catalysts to produce and maintain a set of thermodynamically unstable compounds. This means that the system is maintained away from equilibrium (Pascal et al., 2013). Catalytic networks, or the more abstract concept known as “catalytic task space” (Kauffman, 2000, pp. 13–14), refers to a framework of synchronic systems comprising different reactions, which together form an integrated organization. In other words, the term denotes a group of molecules which affect each other catalytically in order to coordinate the specific places, times, and speeds of their chemical transformations^2^.

As the name indicates, a SM network is a means of preserving the existing organizational structure. Indeed, because they generate a mutual entailment of processes, SM networks tend to persist: they are maintained because of the cyclic nature of the processes that define them. Since, in a SM network, some components make a specific causal contribution to the maintenance of the whole network, variations that do not destroy the organization could in fact be recruited when they contribute to its maintenance. Hence, a SM network tends to incorporate/recruit any contingent organizational or structural modification that may coherently enter it, providing it contributes to its maintenance. Thus, many of the innovations generated among the myriad of reactions occurring in the local environments would have been retained.

However, what is still required is a means of managing the exchange of energy and matter between the organization and its environment. In a prebiotic scenario, the only alternative for achieving this would have been a selectively permeable compartment^3^, which would have helped ensure correct concentrations and would have generated an internal environment which was both differentiated and adjustable in terms of pH, volume and chemical composition, etc. This environment would, in turn, have fostered the development of a metabolic process (and vice versa). The establishment of a division between the internal and external parts of the system would have resulted in concentration gradients (Mitchell, 1961; Harold, 1986), as well as pH and oxidation-reduction differences (Morowitz, 1981, 1992; Chen and Szostak, 2004). These differences may have served as mechanisms for storing energy, perhaps later on forming the basis for endothermic transformations and against gradient active solute transport, which are present in all existing cells (Skulachev, 1992). For these reasons, encapsulation can be seen as a prerequisite for the evolution of a primitive SM organization able to manage the energy flows required to maintain the system (Ruiz Mirazo and Moreno, 2004; Shirt-Ediss, 2016). For its part, the encapsulated organization would have been an instrument for the evolution of a compartment, offering catalysts and other compounds to enable selective permeability to emerge.

Moreover, the formation of self-assembling vesicles and their early association with autocatalytic networks (namely, the formation of “protocells”^4^) may harbor a minimal form of functional variety^5^. For instance, various components of the basic self-maintaining cycle may participate in the generation or composition of the physical border, which in turn would ensure better conditions for recursive self-maintenance. It is also conceivable that the basic self-maintaining cycle would have generated certain components (i.e., catalysts) that would have promoted the processes to follow pathways that were chemically unlikely; or they may have coordinated their inter-conversion processes in space and time in such a way as to ensure a greater number and variety of reactions and components involved in the organization. Thus, to be capable of prebiotic evolution, the organization of a chemical system must have harbored a potential variety of internal constraints which somehow contributed to the system’s self-maintenance (Moreno and Ruiz-Mirazo, 2009; Mossio et al., 2009). This (at least minimal) internal functional differentiation is what is implicit in the concept of a metabolic organization. And this is also the root of a physiological system.

Interestingly, a selectively permeable compartment acts as a global constraint affecting all the internal functions of the system. Indeed, a selectively permeable compartment is able to selectively control transport processes, thereby modifying its constitutive processes to adapt to the environment and ensure the maintenance of its identity. Thus, compartmentalization defines a specific organization in terms of cohesion, physical boundedness and organizational asymmetry with its environment. In sum, since they constitute a specific and physically bounded set of processes, giving rise to a cohesive and functionally integrated organization, self-maintaining protocells realize a minimal form of biological individuality (Moreno, 2016). The capacity to remain in far-from-equilibrium conditions and realize functional differentiation follows on from this cohesive organization.

## The Appearance of Reproduction

In a scenario such as the one described in the previous section, we can conceive of the existence of populations of diverse protocells undergoing processes of fusion and fission, and therefore, of a mechanism of protocell “multiplication.” Of course, this “multiplication” would not have guaranteed organizational similarity; however, it would have permitted a certain degree of diversification and even a minimal form of complexification. Yet, a sustainable process of increase in complexity requires methods of preservation, the reason being that noise will accumulate errors, and since the mechanism ensuring the persistence of the identity of this type of system lies in their organizational circularity, even slight variations would have been deleterious.

The simplest way to overcome this problem is by means of some mechanism allowing organizational redundancy. In other words, it is a mechanism that controls the process of fission so as to guarantee at least a minimal form of organizational repetition. How? If, instead of producing a chaotic form of growth leading to duplication and fission, the internal organization of a type of protocell was able to produce at least a small percentage of new protocells that maintain a sufficient level of organizational similarity with it, the production of an indefinite number of similar organizations would be ensured. This process is reproduction.

The appearance of protocells capable of reproduction (i.e., self-reproducing protocells) would not only have ensured their persistence but would also have rendered them dominant. In a noisy environment that tended to eliminate the less robust forms of protocells (and those slightly more complex ones would most likely have been less robust), only reproduction could have enabled the long-term persistence of the different forms of protocell organization that may have appeared. Despite requiring a more complex organization, due to its inherent capacity to create an indefinite number of spatially separated copies, as well as the fact that it allows some degree of variability, a self-reproducing protocell offers a powerful and robust way of maintaining both past inventions and new innovations.

But how could a minimal form of reproduction have appeared? It is worth to clarify that when we speak here about self-reproducing protocells we do not refer to simple vesicles or other forms of very simple protocells, but of functionally differentiated compartmentalized organizations (as described in the last part of previous section). Whereas the reproduction of the formers is relatively simple (and even, is the usual way they occur in nature), this capacity is instead unlikely in the second case. Not a precise model of a reproduction of such type of a protocell exists yet, but there are different studies showing that a SM protocell can become a self-reproducing organization (and not merely a self-replicative structure) when the ongoing processes of a self-maintenance/production occur in certain specific conditions (Zepik et al., 2001; Solé et al., 2007; Mavelli and Ruiz-Mirazo, 2013; Murtas, 2013; Stano and Luisi, 2016). For example, the deployment of a protocell’s continuous process of self-production may generate growth, and upon reaching a certain size, the compartment (and its contents) may end up in fission, producing two (or more) new similar protocells. And given that the new system and the original one are similar, then, environmental conditions permitting, the process can be reiterated time and time again. Accordingly, reproduction occurs when the ongoing processes of a self-maintaining entity occur in certain specific conditions^6^. In other words, reproduction could only have occurred when a self-maintaining system was organized in a very special way. Let us see now how.

Now, since what we call “growth” implies a process of duplicating certain structures of the system, coordinated with changes in the compartment, a minimal control of the temporal and spatial allocation of the components is also required. This is especially important when the system becomes more complex. For example, when a bacterium reproduces, it triggers fission at just the right moment, ensuring that a set of specific components (not only the genetic ones) are in place to endow each new bacterium with a complete copy of its essential material. However, before fission is achieved, the bacterium must copy many of its components, especially its genetic material, which is segregated and allocated to opposite ends of the system. Next, the different proteins involved in the reproduction process come together at the site where the division is to take place. One vital component of this process is the FtsZ protein, whose monomers arrange themselves into a ring in the middle of the bacterial body, with other components involved in the process then assembling around it. All these elements are positioned in such a way as to ensure that the division divides the cytoplasm without damaging DNA. When division takes place, the cytoplasm splits in two and (in many bacteria) a new cell wall is synthesized. The order and timing of these processes (component replication and segregation, division site selection, invagination of the cell compartment, and synthesis of the new one) are tightly controlled (Weiss, 2004).

Obviously, reproduction would have been much simpler in protocells. It is likely that, when certain parameters were met, simple protocells would have spontaneously settled into a stationary reproducing regime, characterized by regular growth and a division cycle. This would also have involved maintaining a standard size and chemical composition down the generations. As Mavelli and Ruiz-Mirazo (2013) showed, under certain specific conditions, anosmotic synchronization is generated between membrane and core volume growth, and this in turn gives rise to a stationary reproduction regime^7^. In other words, reproduction is not possible without synchronized coordination between the changes taking place in the compartment and the encapsulated SM network.

Of course, reproduction implies also a mechanism that ensures at least a certain degree of similarity between the reproducer and the reproduced. The most primitive form of inheritance would have been probably statistical, namely, only a percentage of the “offspring” would be similar to their ancestors. And only when the earlier genetic components appeared, a mechanism of reliable reproduction could be in place. One possible form of pre-genetic inheritance may be what Segré and co-workers (Segre and Lancet, 2000; Segré et al., 2001) have called “compositional genomes,” which consists in systems that, after duplication, are capable of (statistically) transferring their compositional specificity. More recently, Vasas et al. (2012) and Hordijk and Steel (2014) have presented a model of protocell that could ensure a form of statistical inheritance. Accordingly, even primitive self-maintaining systems lacking template components could be capable of reproduction with a certain degree of identity transmission (although the reliability of the copies would be very low).

All things considered, the appearance of protocells capable of self-reproduction, even in its minimal form, clearly required that these systems be capable of achieving a certain degree of functional integration between all the aforementioned processes, especially between changes in the compartment and changes in the internal network. For all these reasons, reproduction could not have appeared very early on in the process of biogenesis.

## The Paradoxical Nature of Reproduction

As seen above, the appearance of reproduction should be considered the consequence of a very special form of compartmentalized SM organization. However, at the same time, the set of processes that generate reproduction are fundamentally different from those that generate self-maintenance: whereas self-maintenance implies a clear organizational continuity of the same system, reproduction implies a physical discontinuity at the end of the process that connects the reproducing system with the reproduced one. Thus, to what extent can the set of processes that constitute an entire reproductive event be considered an uninterrupted succession of states?

On the one hand, the reproductive process is an actual organization in the following sense: it is a continuous set of processes that progressively build a duplicate organization, with all processes occurring within the same compartment. Let us call this set of processes the *reproducing cycle* (we use the term cycle because the end stage is similar to the initial one). A reproducing cycle is a specific set of processes generated within the temporally larger SM organization of an individuated system, which eventually results in two separate (yet similar) individuated systems. As an actual organization, the reproducing cycle is a far-from-equilibrium concatenation of constraints, each of which functionally contributes to the SM of said organization (in which they operate). Take, for example, the components involved in growth and duplication, or those involved in ensuring adequate temporal control; all these components act as functional constraints, ensuring the correct fulfillment of the reproducing cycle. They act as functional components to the extent that they locally constrain the system’s flows of matter and energy in such a way as to contribute to the maintenance of the global reproducing cycle and, insofar as they themselves are produced within the system, they indirectly exist because of their own action (for more details, see Mossio et al., 2009).

Yet, as mentioned earlier, although the reproducing cycle implies a duplication of the specific SM organization in which it exists, its working is fundamentally supported by this very SM organization: the reproducing cycle itself exists only insofar as it is embodied in the SM (metabolic) organization, which constitutes the individual system^8^. Focusing on the concept of “reproducing cycle” highlights the relevance of an organizational and material connection between parental and filial individual organizations as a necessary mechanism for ensuring sufficiently reliable reproduction. Thus, although in the end the reproducing cycle implies the production of a new, spatially distinguishable system, for reproduction to occur, the underlying organizational continuity between the producing and the produced system(s) cannot be disrupted. This continuity is a necessary condition, because otherwise, as Mossio and Pontarotti (2019) have recently argued, an adequate degree of functional similarity between the producing and the produced systems would not be achieved. And as Griesemer (2002) points out, reproduction is not only the transmission of a “form”; *it* also implies a material connection between the system that reproduces and that which is reproduced. In other words, the similarity between reproducer and reproduced is supported by a material and organizational connection between the metabolic organization of the parental system and that of the filial system.

However, on the other hand, reproduction also implies an organizational discontinuity, because it generates a spatial separation: at the end of the reproducing cycle, part of the set of causal connections occurring in an individuated organization is interrupted and a new, spatially distinct form of individuated organization appears. Gradually, the organization is duplicated, and once the process of duplication and allocation is complete, the border is also duplicated and an organizational discontinuity occurs. Following this disruption, the individual fates of the reproduced and the reproducer, considered as tokens, are different (although, of course, both will be functionally similar).

All these considerations lead us to the following conclusion: in a prebiotic, pre-genetic context, a self-reproducing organization is a specific form of a compartmentalized SM organization, characterized (among other features, like the presence of certain constraints able to control the temporal and spatial distribution of molecules) by the fact that it triggers an indefinite production of similar^9^ (yet spatially separate) organizations. Because of this, each separate entity (*token*) resulting from a reproductive cycle (generation) inherits a specific organizational identity, and the sequence of generations (lineage) therefore constitutes a unique *type*. In a reproductive process, there is type continuity, since there is a mechanism that ensures a similarity between the generator and the generated, and this creates an uninterrupted temporal succession of similar organizational tokens. This is the basis of the *type continuity* between two systems (the reproducer and the reproduced) and, by extension, between all the members of an entire lineage^10^.

The paradoxical fact is that, due to both the maintenance of the organizational continuity of the reproducing cycles and, at the same time, the disruption of this continuity, reproduction generates *another form of continuity*: an organizational *similarity* between an indefinite set of spatially (and temporally) separated protocells. In other words, a self-reproducing organization triggers an indefinite production of similar, yet spatially and temporally separate, organizational tokens. As a result, each separate entity resulting from a reproducing cycle (generation) inherits a specific organizational identity and the sequence of generations (lineage) therefore constitutes a *type*.

The consequence is that, indirectly, reproduction yields an evolutionary history, namely, a causal process that is not apparently based on a continuous entailment of current states. This is the domain of what Mayr (1961) termed “historical causes”. However, as Bickhard (2001) has argued, “history can have causal consequences only insofar as history factors through current state. And appeal to distal causes, such as evolutionary history, is legitimate only insofar as those distal causes factor through current state without loss. Simply put, the past cannot cause anything in the future without the full mediation by the present (state)” (p. 462). Hence, according to Bickhard, we have a problem, since to be causally effective, trans-generation entailments must operate as an entailed set of causal interactions, namely, as an actual organization.

A solution to this problem may be to consider that historical (i.e., trans-generational) causal continuity does not (only) rely on the continuity of the actual organization of the reproducing cycles (which, as we have seen, are interrupted every time a new individual is born). Rather, it exists, fundamentally, because the reproducing cycles ensure a sufficient degree of similarity between the reproduced and the reproducer, such that an indefinite set of iterations of the cycle can be ensured, thus maintaining the *specific form* of organization that reproduces itself and is reproduced. In other words, historical continuity is based on a kind of feedback loop between the actual concatenation of the processes of each reproducing cycle and the type continuity that a reliable iteration of these cycles creates. Thus, reproduction generates a kind of feedback between the “organismal/physiological” and “evolutionary” domains, since the consequence of the iteration of self-reproducing cycles is the long-term continuity of a specific type of SM compartmentalized organization; and the functional role of a particular self-reproducing organization (token) lies in its capacity to trigger a diachronic succession of similar self-reproducing organizations, i.e., a lineage. A lineage, in turn, contributes to the maintenance of a specific type of self-reproducing organization.

The specific functional components involved in the accomplishment of a self-reproducing cycle do not contribute directly to the token organization in which they operate, but are retained because, indirectly (i.e., through the iteration of reproductive cycles), they contribute to creating and maintaining intergenerational similarity, which in turn, (and again indirectly) contributes to the maintenance of the very specific type of self-reproducing organization in which they are generated^11^. In other words, to be functional, the structures involved in the reproductive cycle of an individuated organization require the establishment of an intergenerational lineage, namely, the participation of successive different (yet organizationally similar) tokens.

This entangled relationship between reproducing cycles and their consequences is the basis for the unfolding of evolutionary historicity. This, in turn, generates a completely new set of conceptual categories: populations, lineages, selection^12^, fitness landscape, etc., which together define what is termed “the evolutionary domain”. Next, we will analyze the consequences of the origin of this entangled relationship.

## The Consequences of Reproduction: The Appearance of a Multidimensional Organization

As we have seen, even before the appearance of genes, self-reproduction with some form of inheritance led to intergenerational entailments between protocells. These entailments are the basis of a very primitive form of evolution, understood as the intergenerational change of populations.

According to this view, the two dimensions (the physiological and the evolutionary) are not symmetrical. Whereas the physiological dimension appears as an actual SM organization, the evolutionary domain appears as an unfolding of a mechanism, full of contingent events. Although, strictly speaking, an evolutionary perspective is not only a diachronic view, because it implies also a continued process of selection between synchronically competing phenotypes, it is usually understood as a historical phenomenon: Thus, evolutionary Biology is essentially focused on the study of changes in the heritable characteristics of reproducing sets of organisms (and even proto-organisms) over successive generations (Hall and Hallgrimason, 2008). And, given that to a large extent these changes are contingent, evolution is also, largely, a historical field of study.

However, the appearance of populations and lineages also created a new synchronic domain, this time operating at a different temporal (and spatial) scale than that of individuated protocells. This domain appeared as a consequence of the long-term action of different communities of metabolically similar protocells modifying their collective environment. Thus, these different sets of protocells may have generated mutually self-sustaining and complementary interactions.

Of course, this would have required several conditions to be met: first, the existence of stable communities at a long-term temporal scale (compared with the short-term scale of the protocell’s lifespan), and therefore of reproduction with a minimal form of inheritance; and second, a transition from a domain where the number of different types of individualities was very small to another where the variety of types increased considerably (which in turn is linked to an increase in the reproductive reliability of the individualities). In other words, at any given time, the combination of individual action and evolutionary processes generated communities of different types of protocells which mutually and synchronously affected their environmental conditions.

Because of their similar metabolic identity, each of these different types of communities would have collectively modified their environment, together constraining the flows of matter and energy in a specific way; and when these different flows of matter and energy found a complementary relationship, they became stabilized and the communities created the conditions required to ensure their global long-term maintenance. Thus, although evolution (based on heritable reproduction) laid the groundwork for the appearance of increasingly complex individuated prebiotic organizations, the emergence of these collective self-maintaining webs is another equally important prerequisite for the long-term sustainability of the process of biogenesis.

These collective SM webs can be seen as constituting the earlier forms of an ecological domain. Odenbaugh (2010) defines the essence of an ecosystem in terms of energy flows and biogeochemical cycles involving components of both a biotic and abiotic nature, stating that: “an ecosystem exists just in case biotic and abiotic members of a set are closed under these ecosystemic causal relations” (p. 245). What the ecological perspective introduces is an intrinsically cooperative and systemic approach, where the actors of the relevant interactions are collections of (reproductive) physiological individuals, which together construct an environment (niche construction). Moreover, whereas selection has often been seen as an action carried out by the environment on almost passive organisms, niche construction is an active process performed by specific collections of organisms^13^. The systemic dimension of ecological interactions lies in the complementary functionality of the different communities in a given ecosystem, which together constitute a global self-maintaining network (Nunes-Neto et al., 2014). What defines ecological interactions is the fact that an action carried out by a particular kind of organism (or proto-organism) on a particular environment has an effect on the inflow of energy and material pertaining to another type of organism, and this, in its turn, carries out an action which affects another group, and so on, until the folding up of the entire set of interactions. This closed network of interactions constitutes a far-from-equilibrium SM organization.

The importance of the appearance of ecological networks lies in the fact that they enabled the long-term sustainability of prebiotic systems in both energy and material terms. The action carried out by the different types of (proto)organism guaranteed the constraint of the energy and matter flow required for both their own maintenance and that of other types of (proto)organisms, thereby ensuring their indefinite maintenance (provided that certain geological and astronomical conditions were met: for example, ultimately, the network had to be driven by a stable external energy source).

Ultimately, of course, each of these different constrained flows of energy would have been based on the “microscopic” environmental action of long-term stable communities of similar individuated protocells. Globally, the creation of stable ecological systems would in turn have helped to ensure the long-term maintenance of differentiated protocells, since it enabled them to sustain not only evolution but also the environmental conditions necessary to develop more complex metabolic organizations. This is why an ecological system became, as Dagg (2003) points out, a kind of “biologically constructed environment”.

It is certainly difficult to determine in which stage of prebiotic evolution a primitive form of ecosystem may have appeared. Even at the level of very simple artificial protocells, some experiments have observed the development of “colonies” which might have facilitated vesicle fusion and solute capture, generating a positive feedback loop between “individual” systems and the “colony” (Carrara et al., 2012; Stano et al., 2014). Since this interdependency between the individual and the colony may be observed even in the simplest of protocells, during later phases, this interaction would probably have undergone a process of reinforcement and complexification. It is therefore sensible to hypothesize that, as self-reproducing^14^ protocells became more metabolically complex^15^ and capable of producing more complex compounds within themselves, a fairly dense group of said compounds would have begun to accumulate in their vicinity, with some being harnessed and used by other protocells, providing, of course, they were functional for them. This would have led to one of two outcomes: a dead end, resulting from the ongoing rise in the quantity of protocells and scarcity of resources in the surrounding area; or the generation of a network of metabolic dependencies among the individuated systems. Furthermore, as Guerrero (1995), Guerrero et al., 2002), and Ruiz-Mirazo et al. (2004) have argued, this proto-metabolic complementarity among different kinds of protocells would have eliminated the need to “clean” up the growing quantity organic waste that could not be digested. Similarly, and more recently, Briones et al. (2015) stated that:

“Thanks to the fundamental connection between the membrane and the metabolism, a continuous flow of energy and matter between each system and its environment began to occur. This would have prompted a movement of substances between different protocells living in close proximity, which is essential because each compartmentalized system would have been slightly different from the others, and none would have been able to produce all the molecules needed. Moreover, lacking mechanisms for the reuse of certain basic chemical compounds, sooner or later a global crisis would have occurred due to depletion of available resources. Therefore, moving on from their earlier steps, groups of protocells began to establish ecological relationships with each other: the beginning of life not only marked the beginning of evolution, but also the beginning of ecology.” (p. 266, our translation).

Thus, the origin of ecosystems can be traced back to a scenario in which a certain *community* of protocells would have affected the inflow of energy and material pertaining to another community of protocells, whose own metabolic impact on its environment would have in turn affected another group of protocells, with the cycle repeating itself until the loop was closed. As a result, a web of metabolic dependencies would have been established among them. Hence, over time, different types of populations would have established functional interactions and relatively stable collective networks.

This self-maintaining network of (proto)ecological interactions is important because, while said interactions are the result of long-term metabolic interactions among a diversified community of protocells, the (proto)ecosystem also enables the sustainability of this community of protocells in the long term, in terms of both energy and materials. Seen from this global perspective, the ultimate consequence of the appearance of reproduction in biogenesis was the creation of a new *self-maintaining organizational* domain, operating at a much broader temporal and spatial scale than that of the physiological domain of proto-organisms. Long-term stable groups of individuated self-reproducing systems, characterized by their different forms of metabolism, created the mechanisms and interactions that allowed the emergence of a long-term (yet synchronic) macroscopic self-maintaining network (an ecosystem). From this perspective, evolution is the means by which individual entities became ecological communities, and the mechanism that allowed the progressive plasticity and diversity of such communities.

## Concluding Remarks

In this paper, we have tried to show that understanding the origin of life requires the explanation of the early appearance of a new form of circular causality in the process of biogenesis, articulated at different spatial and temporal scales. This multilevel form of causality, in turn, is based on the appearance of reproduction, which, being itself the expression of a special form of SM organization endowed with a form of cohesive individuality, permitted at the same time a cumulative historical process and the generation of synchronic collective SM ecological networks.

In this context, we should reconsider the concept of “actual organization.” As we have argued, reproduction creates a temporal multi-scale organization, thereby implying (at least) two different classes of current states: those happening at the temporal scale of individuated systems and those happening at the temporal scale of the ecosystem. And in a multiscale organization such as this, short-term processes are in turn affected by slower and spatially larger processes. Also, in this context, a type is not a purely abstract entity; rather it occupies a certain space (although not continuous) and endures over a period of time.

At the lowest level, this form of organization is represented by cells (or protocells), which are spatio-temporally bounded organizations, and could be considered as individuated identities. This form of organization is also the most basic and the first to appear. Their organizational identity is a *token* identity. But when they reproduce, they generate an organizationally discontinuous set of similar entities, which in turn will generate a spatio-temporally broader form of organization: an evolving ecosystem. So, if one changes the time-space scale, a former type could be seen as a token, and thus, the type-token distinction appears as scale dependent. In a similar vein, therefore, the concept of “current organization” is also scale dependent, and what actually matters is the connection created between different scales.

Of course, processes occurring within protocells are far from the hyper complex physiological processes that constitute present-day organisms; and, similarly, the pre-Darwinian (and proto-ecological) processes here sketched are much simpler than those happening in the biological world. But what I am arguing is that the proto-metabolic organization of these types of protocells *points to* what in a full-blown biological organism is a physiological organization; and that their reproductive dynamics, similarly, *foreshadows* the evolutionary and population dimension of life.

If this interpretation is right, the physiological domain has its roots in the constitutive self-production of the earlier localized entities that will progressively become organisms. But this path will require that at the same time a class of these primitive entities develop a reproductive capacity and as a consequence, they will generate a fist form of a multiscale phenomenon, that expressed itself in the form of individuated, cohesive systems (proto-organisms), as well as in collective, physically unbounded networks (proto-ecosystems; the whole biosphere); in actual organizations (self-maintaining metabolisms, food-webs), and in causally correlated sets of diachronic/historical processes (represented by phylogenetic trees). Thus, even before the appearance of a full-fledged biological domain, biogenesis would have enacted an entangled multidimensional and multiscale self-maintaining system that was both individual and collective; synchronic and diachronic. And it is due to the generation of this multiscale organization that prebiotic evolution will progress and become a robust process.

## Author Contributions

The author confirms being the sole contributor of this work and has approved it for publication.

### Conflict of Interest

The author declares that the research was conducted in the absence of any commercial or financial relationships that could be construed as a potential conflict of interest.

The reviewer CM declared a past co-authorship with AM.

## Footnotes

^1^This is a key question in relation to the Origins of Life because a chemically stable and diverse environment would, in turn, enable individual systems with a lower level of metabolic complexity. As argued by both Morowitz (1992) and Morange (2008), metabolic simplicity is dependent on how chemically demanding the environment is. A changeable and chemically restricted environment would be more demanding than a very stable and chemically rich one. Nevertheless, no one has, to date, clarified the exact nature of the relationship between environmental and metabolic complexity in early evolutionary stages. Another issue linked to the stability of far-from-equilibrium chemical organizations is the interaction between individuated systems and collective networks, defined as sets of different types of localized systems in constant interplay with environmental compounds, which together constitute a stable kind of collective self-maintaining system. We will address this question in Section “The Consequences of Reproduction: The Appearance of a Multidimensional Organization.”

^2^More technically, this idea of SM network is similar to that of a “reflexively catalytic network,” a more abstract concept that denotes a series of cyclic reactions in which each reaction is driven by one (or more) module from one of the other reactions. Furthermore, all reactants within the system are generated from a small central group of components (Hordijk and Steel, 2004). Although such a system has yet to be reproduced *in vitro*, various theories postulate that they may have emerged prior to the RNA stage (Hordjik et al., 2012; Vasas et al., 2012). See also next section.

^3^Prebiotic compartments would likely have emerged due to the spontaneous assembling of amphiphilic molecules in water (Deamer, 1985; Deamer and Dworkin, 2005).

^4^The term “protocell” is used to denote any experimental or theoretical model involving a self-assembling compartment connected to chemical processes taking place around or within it (Rasmussen et al., 2008; Ruiz-Mirazo, 2011). Here, the term is used slightly more specifically, referring to a compartmentalized system that is both far-from-equilibrium and self-maintaining, and which has some prebiotic properties, including growth, autocatalytic activities, and reproduction.

^5^As argued by Bickhard (2000), Mossio et al. (2009), and Mossio and Moreno (2010), a system shows functional diversity if it is a dissipative SM organization made up of a series of structures (catalysts, membrane, etc.) which constrain energy flows in such a way that they maintain the system where they exist and thus reproduce themselves.

^6^Actually, rather than a form of self-maintenance/production, reproduction constitutes *a duplication*: while SM is a temporally indefinite repetition of a localized unique form of organization, (self)reproduction is an indefinite spatial propagation of a similar organization. As a consequence, whereas in a continuous process of SM, the eventual variations lead to the forgetting-erasing of the initial organizational identity, in a process of reproduction, variations may occur in some reproductive sequences, while others maintain the previous identity. Moreover, SM does not propagate, while reproduction does, thanks to an organizational discontinuity between the reproducer and the reproduced, which occurs in the form of a spatial discontinuity resulting from the achievement of a growth process ending in (at least) two similar organizations.

^7^Thus, even the simplest forms of self-maintaining organizations, which have no template components, can transmit a certain amount of identity during reproduction (although the fidelity of the copies would, of course, be very low).

^8^Moreover, reproduction implies a cost for the standard functioning of the basic metabolic organization (because it makes no contribution to the actual system in which it operates). In operational terms, a reproducing SM system can survive/persist indefinitely only because reproduction is a cycle that (sooner or later) ends, and the system recovers its standard metabolic regime. However, from a broader perspective, this “dysfunctional” regime (at the level of the individuated system) is the way by which intergenerational causes could operate.

^9^Similarity is ensured by organizational continuity (as we have explained) and by the transmission of genetic materials. Although the appearance of each new individual (generation) implies a complete renewal of the material elements, this renewal occurs without interrupting the individual’s “basic” (lineage) identity, thanks to both organizational continuity and the stability of the hereditary constraints.

^10^A lineage is a series of generations derived from an ancestral genetic type. In other words, it is a temporal series of organisms connected by a continuous line of descent from ancestor to descendent. In a pre-biotic pre-genetic context, a lineage is more difficult to define; but we can say that a temporal series of self-reproducing protocells, which are connected by a continuous line of descent from ancestor to descendent, and which maintain a certain degree of functional identity, could be considered a primitive form of lineage.

^11^Actually, the chain of causes and effects forming this causal loop is more complex. The dynamic effects between each protocell and its environment (which is of course also constituted by other protocells and their side products) affect its offspring and therefore slowly shape the type of inherited components received by each new generation of protocells (and vice versa).

^12^Selection here means the process whereby self-reproducing protocells that generate more offspring will prevail at the expense of other types of protocells. Notice that this concept of selection is trans-generational (and therefore diachronic). It is therefore different from that of strictly synchronic competition which depends on the more or less successful management of environmental conditions by different types of protocells, since selection in this case will occur regardless of reproduction. Nevertheless, a synchronic disadvantage will usually lead to a diachronic disadvantage also.

^13^For the purpose of the argument, a self-reproducing protocell can be considered equivalent to a very primitive organism (see below). Hence, in this case, communities of protocell lineages can be considered “biotic” entities which may, under certain circumstances, achieve the ecological relationships defined here.

^14^Because trans-generational continuity (i.e., lineages) is a requirement for the emergence of an ecological domain.

^15^Especially after the appearance of the earlier genetically based proto-organisms.
